# Functional analysis of the mating type genes in *Verticillium dahliae*

**DOI:** 10.1186/s12915-024-01900-6

**Published:** 2024-05-07

**Authors:** Ya-Duo Zhang, Xiao-Bin Ji, Juan Zong, Xiao-Feng Dai, Steven J. Klosterman, Krishna V. Subbarao, Dan-Dan Zhang, Jie-Yin Chen

**Affiliations:** 1 State Key Laboratory for Biology of Plant Diseases and Insect Pests, Institute of Plant Protection, Chinese Academy of Agricultural Sciences, Beijing, 100193 China; 2 United States Department of Agriculture, Agricultural Research Service, Salinas, CA USA; 3 Department of Plant Pathology, University of California, Davis, c/o United States Agricultural Research Station, Salinas, CA USA; 4 Western Agricultural Research Center, Chinese Academy of Agricultural Sciences, Changji, 831100 China

**Keywords:** *Verticillium dahliae*, Mating type, Pheromone, Sexual reproduction, Asexual reproduction, Virulence

## Abstract

**Background:**

Populations of the plant pathogenic fungus *Verticillium dahliae* display a complex and rich genetic diversity, yet the existence of sexual reproduction in the fungus remains contested. As pivotal genes, *MAT* genes play a crucial role in regulating cell differentiation, morphological development, and mating of compatible cells. However, the functions of the two mating type genes in *V. dahliae*, *VdMAT1-1-1*, and *VdMAT1-2-1*, remain poorly understood.

**Results:**

In this study, we confirmed that the *MAT* loci in *V. dahliae* are highly conserved, including both *VdMAT1-1-1* and *VdMAT1-2-1* which share high collinearity. The conserved core transcription factor encoded by the two *MAT* loci may facilitate the regulation of pheromone precursor and pheromone receptor genes by directly binding to their promoter regions. Additionally, peptide activity assays demonstrated that the signal peptide of the pheromone VdPpg1 possessed secretory activity, while VdPpg2, lacked a predicted signal peptide. Chemotactic growth assays revealed that *V. dahliae* senses and grows towards the pheromones FO-a and FO-α of *Fusarium oxysporum*, as well as towards VdPpg2 of *V. dahliae*, but not in response to VdPpg1. The findings herein also revealed that *VdMAT1-1-1* and *VdMAT1-2-1* regulate vegetative growth, carbon source utilization, and resistance to stressors in *V. dahliae*, while negatively regulating virulence.

**Conclusions:**

These findings underscore the potential roles of *VdMAT1-1-1* and *VdMAT1-2-1* in sexual reproduction and confirm their involvement in various asexual processes of *V. dahliae*, offering novel insights into the functions of mating type genes in this species.

**Supplementary Information:**

The online version contains supplementary material available at 10.1186/s12915-024-01900-6.

## Background

Sexual reproduction is a ubiquitous characteristic of eukaryotes, which can combine elite alleles from different individuals and repair random epigenetic or conventional genetic damage through the processes of crossover and recombination during meiosis [21, 22, 48]. In contrast to sexual reproduction, strictly asexual reproduction is often considered as an evolutionary dead-end, mainly because there is no meiotic recombination, leading to an increase in the accumulation of harmful mutations [41, 56]. These effects are known as Muller’s ratchet [24, 43]. Approximately, 20% of all species of fungi are considered strictly asexual, without a recognized sexual cycle, and in the Ascomycota, the rate is higher, at up to 40% [56]. However, the sexual cycle of some species may be not absent, but rather cryptic since the hallmarks of a sexual cycle have not been directly observed [25, 35, 47].

In the fungal kingdom, sexual reproduction has evolved into two sexual breeding systems, and these systems are homothallism or heterothallism. Sexual reproduction in homothallic ascomycetes is being self-fertile; however, in heterothallic ascomycetes, mating occurs only between two different strains belonging to opposite mating types [1, 39]. In heterothallic fungi, the determination of mating type is governed by the presence of idiomorphic alleles *MAT1-1* and *MAT1-2*. Conversely, homothallic species possess genes for both *MAT* loci, which may be situated either at a singular *MAT* locus or on distinct chromosomes [45]. The mating type locus encodes transcription factors that determine mating type identity and serve as master regulators of sexual reproduction [55]. The *MAT1-1* locus encodes an α-domain transcription factor named as *MAT1-1-1*, while its idiomorph, the *MAT1-2* locus, encodes a high mobility group (HMG) transcription factor referred to as *MAT1-2-1* [38, 57]. *MAT* genes are critical for regulating cell differentiation, development, and mating of compatible cells. In addition, *MAT* genes also play important roles in fungal vegetative growth, conidial morphology, conidiospore germination, mycelial development, amino acid, secondary, and iron metabolisms [5, 8, 36].

A vital step in the early stages of sexual reproduction among heterothallic fungi is mate recognition, which depends on the pheromone systems of strains with opposing *MAT* loci. One fungal strain secretes pheromones that bind to heptahelical pheromone receptors expressed by the strain with opposite *MAT* type. The interaction between pheromone and its receptor triggers the G-protein-regulated signal transduction and induces the expression of mating-related genes, causing chemotactic growth of strains towards cells of the opposite mating type [3]. Pheromone precursor genes have been identified in several heterothallic filamentous ascomycetes, including *Gibberella zeae*, *Sordaria macrospora*, and *Neurospora crassa* [7, 37, 42]. In these ascomycetes, one of the genes encodes a pheromone polypeptide with a C-terminal carboxy methyl isoprenylated cysteine, which is derived from pheromone precursor with C-terminal CaaX (C, cysteine; a, aliphatic; and X, any amino acid residue) motifs. The other precursor genes encode a polypeptide containing multiple repeats of a putative pheromone sequence bordered by protease processing sites [51]. Deletion of pheromone receptor *pre1* in *N. crassa* causes female sterility [30], while deletion of either pheromone precursor gene causes male sterility, as spermatia could no longer attract female trichogynes [31]. In *Gibberella zeae*, Δ*ppg1* reduced male fertility and Δ*pre2* reduces female fertility in outcrossing tests [37]. Similarly, in *S. macrospora*, the absence of any compatible pheromone receptor pair (Δ*pre2*/Δ *ppg2*, Δ*pre1*/Δ*ppg1*) and the double-pheromone mutant (Δ*ppg1*/Δ*ppg2*), results in a significant reduction in the number of perithecia and sexual spores. Moreover, the deletion of both receptor genes (Δ*pre1*/Δ*pre2*) prevents the formation of fruiting bodies and ascospores [42].

*V. dahliae* is a soilborne plant pathogen that invades and colonizes the xylem tissue, resulting in Verticillium wilt diseases on over 200 plant species [20, 34]. *V. dahliae* has a complex population structure, comprising physiological races 1, 2, and 3 [10]; defoliating (D) and nondefoliating phenotypes (ND) [66]; vegetative compatibility groups (VCGs) [6, 13, 14, 29], and clonal lineages [4, 18, 44, 53]. In addition, *V. dahliae* has two mating types, *MAT1-1* and *MAT1-2*, and has maintained all the machinery required for sexual reproduction [53], but whether it undergoes sexual recombination has been a controversial topic [44, 53, 54]. Some reports suggest that *V. dahliae* may reproduce strictly asexually as it has a clonal population structure, with little genetic variation between strains of the same clonal groups and there is weak evidence for recombination [2, 52]. In addition, the distribution frequencies of these two mating type strains *MAT1-1* and *MAT1-2* differ significantly in *V. dahliae* population (with < 1% of sampled strains carrying the *MAT1-1* idiomorph) [54]. Therefore, the probability of sexual reproduction between individuals of opposite mating type in *V. dahliae* in nature is low. Moreover, the genome of *V. dahliae* has undergone chromosomal rearrangements that may interfere with meiosis and reduce the probability of successful sexual reproduction between existing lineages [15, 46]. Lastly, no fructifications from sexual reproduction, such as the apothecia, perithecia, or cleistothecia, have been found in *V. dahliae*, either in nature or in the laboratory. In contrast, the possibility that *V. dahliae* undergoes cryptic sexual reproduction cannot be discounted as the generation of new clonal lineages is suggestive of sexual recombination [13, 14, 29]. In addition, the evolutionary relationship between different lineages is closer than the relationship between two distinct lineages derived from the same lineage, a pattern that could be explained more parsimoniously by recombination rather than mutation [44]. Also, each isolate of *V. dahliae* contains either the *MAT1-1* or *MAT1-2* idiomorph, indicating that *V. dahliae* is heterothallic [58, 59], and many sex-related genes that are necessary for the sexual cycle in other fungi are conserved in *V. dahliae* [54]. *V. dahliae* is one of the parents of *V. longisporum*, a hybrid species derived from three separate hybridization events [27]. Thus, *V. dahliae* may have a cryptic sexual cycle or has had an ancestral sexual lifestyle.

*MAT* genes play a pivotal role in fungal sexual reproduction, as they are essential for regulating cell differentiation, development, and mating of compatible cells. *V. dahliae* populations possess two mating type strains, *MAT1-1* and *MAT1-2*, but the functions of the mating type genes *VdMAT1-1-1* and *VdMAT1-2-1* remain largely unexplored. The crucial step in the initial phase of fungal sexual reproduction is mate recognition, which depends on the strain’s pheromone system. Yet, the roles of the pheromone precursor genes and pheromone receptor genes in *V. dahliae* are still not well understood.

In the present study, we characterized the mating type genes *VdMAT1-1-1* and *VdMAT1-2-1* in *V. dahliae*. Our results revealed VdMAT1-1-1 and VdMAT1-2-1 possess the capability to bind directly to the promoter regions of pheromone precursor and receptor genes, thereby exerting regulatory control over their expression. Subsequently, we demonstrated that *V. dahliae* exhibits chemotactic behavior by sensing and orienting growth in response to pheromone signals. Finally, our findings indicated that *VdMAT1-1-1* and *VdMAT1-2-1* are integral not only to the pathogen’s ability to adapt to varying environmental conditions but also play a crucial role in its vegetative growth and pathogenicity. These insights contribute significantly to our understanding of the molecular mechanisms that underpin pathogenicity and complex regulatory processes in *V. dahliae*.

## Results

### Identification of the *MAT* loci *V. dahliae*

**Fig. 1:**
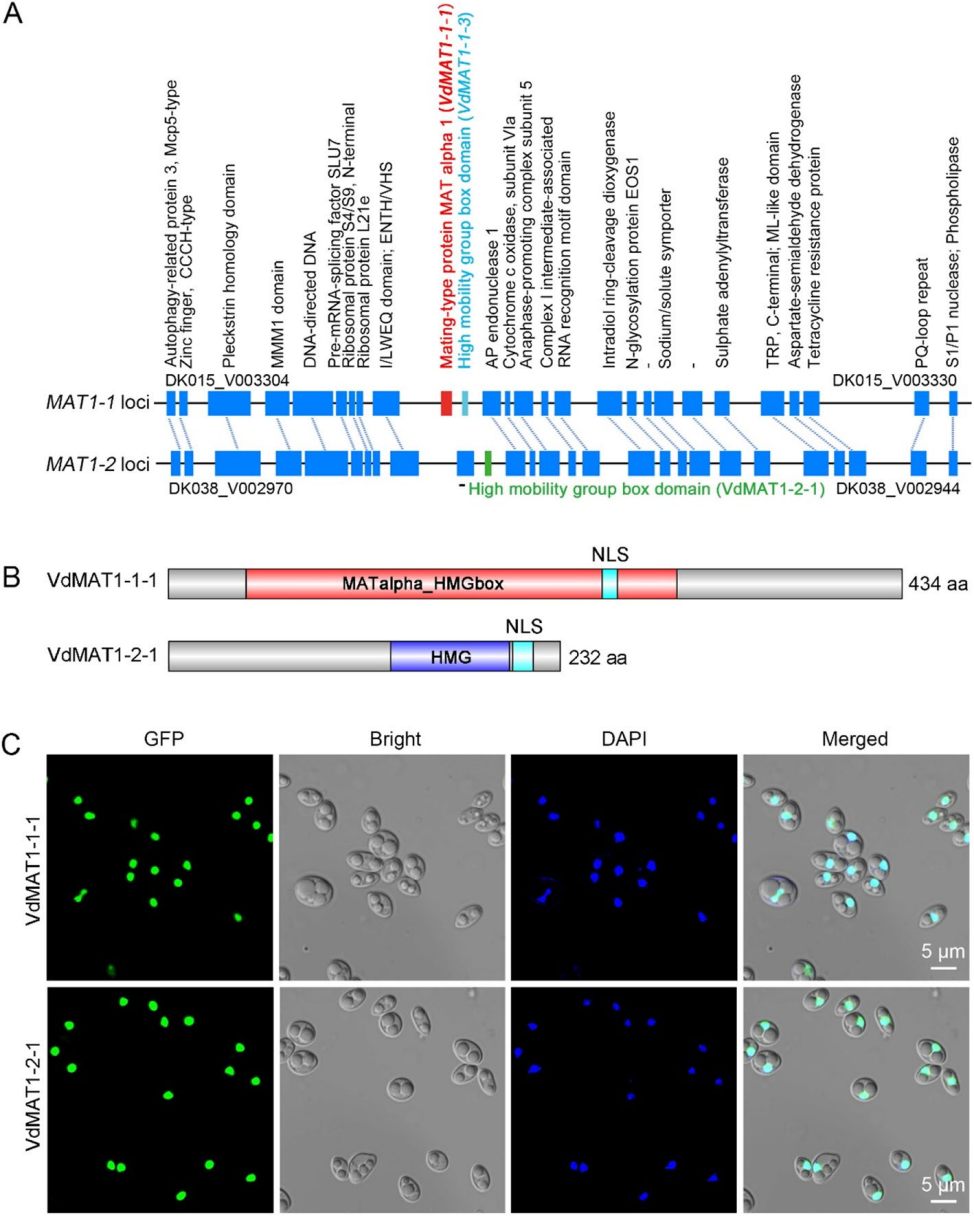
Characteristics of two *MAT* loci in the *Verticilium dahliae* strains DK015 and DK038 of opposite mating type. **A** Diagram of *MAT1-1* and *MAT1-2* loci and flanking sequences in *the MAT1-1* strain DK015 and the *MAT1-2* strain DK038. Rectangles represent the genes within the two loci. The dash line indicates the syntenic relationship between two homologous genes flanking both loci. The text above describes the functional annotation of each gene, with the gene IDs of the first and last gene marked. Red and bright light blue rectangles indicate genes specific to *VdMAT1-1* locus, *VdMAT1-1-1*, and *VdMAT1-1-3*, respectively, while the green rectangle indicates the gene specific to *VdMAT1-2* locus, *VdMAT1-2-1*. Short vertical lines indicate that the gene function has not been annotated. **B** Structure schematic of VdMAT1-1-1 and VdMAT1-2-1 proteins. The red column represents the MATα_HMG box in VdMAT1-1-1. The purple column represents the HMG domain in VdMAT1-2-1. Light blue, nuclear localization signal (NLS). Gray boxes represent unknown protein structure. Aa, amino acid. HMG, high mobility group. **C** Subcellular localization of VdMAT1-1-1 and VdMAT1-2-1 fused with GFP proteins in *V. dahliae* conidia. The nuclear signal was confirmed with the aid of 4, 6-diamidino-2-phenylindole (DAPI), a nuclear dye. The expressed GFP fusions were excited with a 484 nm wavelength, with the emission captured at 507 nm wavelength. DAPI was excited at 360 nm, captured at an emission wavelength of 460 nm. Bars = 5 μm

The strains DK015 and DK038, with opposite *MAT* loci, were identified from the Verticilli-Omics project. Whole genome comparative analyses indicated that DK015 and DK038, isolated from spinach seeds, had a similar karyotype with very little chromosome rearrangement, and shared 94.6% (9739 orthologs) orthologous genes (unpublished data). DK015 and DK038 contained the *MAT1-1* and *MAT1-2* idiomorphs, respectively (Fig. 1A). Both *VdMAT1-1* and *VdMAT1-2* loci and the corresponding flanking sequences were aligned. The coding genes within the flanking sequences of the two *MAT* idiomorphs are highly conserved, such as the APN2 (AP endonuclease 2), COX13 (Cytochrome c oxidase, subunit VIa), and APC5 (Anaphase-promoting complex subunit 5). The genes coded by the two *MAT* loci are markedly different, with the *MAT1-1* locus containing *VdMAT1-1-1* and *VdMAT1-1-3* and the *MAT1-2* locus containing *VdMAT1-2-1* (Fig. 1A). In addition, *MAT* idiomorphs of other *MAT1-1* and *MAT1-2* strains isolated from tomato, potato, cotton, sunflower, and watermelon were also analyzed. The results were similar to those of DK015 and DK038 strains, indicating a high conservation of the *MAT* idiomorphs in *V. dahliae* (Additional file 1: Fig. S1).

Cloning and sequence analysis confirmed that *VdMAT1-1-1* encodes a protein with 434 amino acids containing a MATalpha_HMGbox domain, while *VdMAT1-2-1* encodes a protein with 232 amino acids containing an HMG domain (Fig. 1B). Both VdMAT1-1-1 and VdMAT1-2-1 contain one nuclear localization signal (NLS) that was consistent with their conventional function of regulating the expression of downstream genes. Correspondingly, subcellular localization results verified that both GFP-fused MAT proteins were located in the nucleus (Fig. 1C).

### VdMAT1-1-1 and VdMAT1-2-1 regulate the expression of pheromone and pheromone receptor genes by directly binding to their promoter regions

**Fig. 2:**
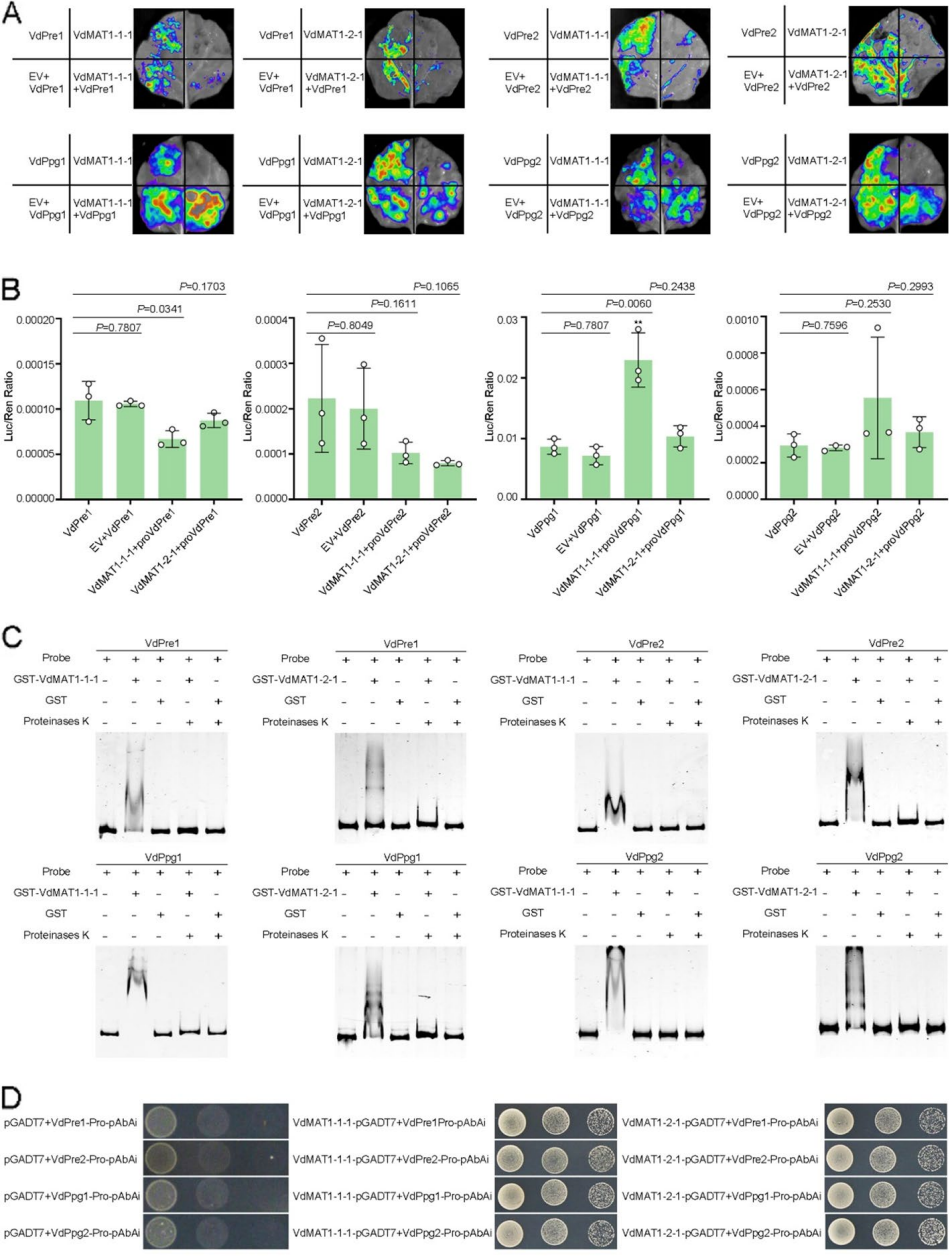
*Verticillium dahliae* VdMAT1-1-1 and VdMAT1-2-1 regulate the expression of pheromone precursors and receptor genes by binding to promoter motifs. **A**, **B** Dual-luciferase reporter assays of VdMAT1-1-1 and VdMAT1-2-1 and their ability to regulate the expression of *VdPpg1*, *VdPpg2*, *VdPre1*, and *VdPre2*. **C** Electrophoretic mobility transfer assay (EMSA) for the analysis of VdMAT1-1-1 and VdMAT1-2-1 binding to the promoter regions of the *VdPpg1*, *VdPpg2*, *VdPre1*, and *VdPre2* genes. A shifted band was observed when purified glutathione S-transferase (GST)-tagged VdMAT1-1-1/VdMAT1-2-1 protein were co-incubated with promoter fragments of *VdPpg1*, *VdPpg2*, *VdPre1*, and *VdPre2*, respectively. After the addition of proteinase K, the hysteretic band disappeared, leaving only the free probe visible. When GST protein were co-incubated with promoter fragments of *VdPpg1*, *VdPpg2*, *VdPre1*, and *VdPre2*, no shifted band occurred. **D** Yeast one hybridization (Y1H) experiment confirmed that VdMAT1-1-1 and VdMAT1-2-1 could directly bind the [5′-ATTGA-3′] motifs in the promoter region of *VdPpg1* and *VdPre2* genes, and the [5′AACAAT-3′] motifs in the promoter region of *VdPpg2* and *VdPre1* genes

A key role of *MAT* gene products in heterothallic species is to regulate the expression of the pheromone signaling system, which is involved in the recognition of mating partners [3]. The expression of pheromone precursor and pheromone receptor genes are directly controlled by MAT transcription factors [17]. To investigate the roles of VdMAT1-1-1 and VdMAT1-2-1 in regulating the pheromone (*VdPpg1* and *VdPpg2*) and pheromone receptor genes (*VdPre1* and *VdPre2*), we first performed the dual-luciferase reporter assay in *N. benthamiana*. The results indicated that VdMAT1-1-1 and VdMAT1-2-1 could inhibit the transcription activity of the *VdPre1* and *VdPre2* promoters, while enhancing the transcription activity from the *VdPpg1* and *VdPpg2* promoters (Fig. 2A, B). Electrophoretic mobility shift assay (EMSA) further demonstrated that both VdMAT1-1-1 and VdMAT1-2-1 could directly bind to the promoter regions of *VdPpg1*, *VdPpg2*, *VdPre1*, and *VdPre2* (Fig. 2C). In addition, sequence analysis revealed a conserved binding motif, 5′-AACAAT-3′, within the promoter regions of *VdPpg2* and *VdPre1*, which exhibited interactions with both VdMAT1-1-1 and VdMAT1-2-1 (Fig. 2D). Additionally, direct interactions between VdMAT1-1-1 and VdMAT1-2-1 and the *VdPpg1* and *VdPre2* promoter regions were substantiated through yeast one-hybrid (Y1H) assays with VdMAT1-1-1 and VdMAT1-2-1 and the conserved sequence motif 5′-ATTGA-3′ found upstream of the *VdPpg1* and *VdPre2* coding regions (Fig. 2D). These results indicate that VdMAT1-1-1 and VdMAT1-2-1 regulate the expression of *VdPpg1*, *VdPpg2*, *VdPre1*, and *VdPre2* genes by directly binding to their promoter regions.

### *V. dahliae* strains perceive pheromones and exhibit chemotactic growth

**Fig. 3:**
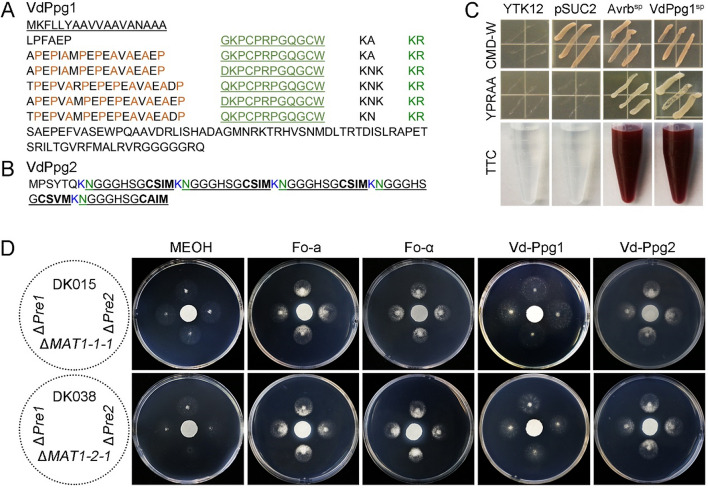
Functional analyses of peptide pheromones from *Verticillium dahliae*. **A** The amino acid sequences of the Ppg1-like peptide pheromones from *V*. *dahliae*. Repeated parts are highlighted in green font and underlined. Kex2 processing sites (KR) are indicated in green, with putative STE13 processing sites in brown. The hydrophobic leader sequence is underlined. **B** The amino acid sequence of the Ppg2-like peptide pheromone from *V*. *dahliae*. Repeated parts are highlighted with a black underline. Lysine residues and asparagine residues of the putative processing sites are indicated in blue and green, respectively. The C-terminal CaaX farnesylation motif and internal Caax-like motifs are bolded. **C** Functional validation of the signal peptide of VdPpg1 by a yeast signal trap assay. The region encoding the 18 aa *N*-terminal peptide of VdPpg1 was fused in-frame to the invertase sequence in the pSUC2 vector and transformed into yeast strain YTK12. The signal peptide of the oomycete effector Avr1b was used as a positive control. The untransformed YTK12 strain and the YTK12 strain carrying the empty pSUC2 vector were used as negative controls. The yeast strain YTK12 cannot grow on a CMD-W medium without tryptophan, whereas strains containing pSUC2 vector could grow based on the function of the Trp operons. Only yeast strains that secrete invertase can convert 2, 3, 5-triphenyltetrazole chloride (TTC) to red triphenylformazan. If the yeast strain can secrete the invertase, it can convert 2, 3, 5-triphenyltetrazole chloride (TTC) into red triphenylmethylzan. **D** Test of chemotactic pheromone-responsive growth of *V. dahliae*. MEOH served as a negative control while FO-a and FO-α are a-like and α-like peptide pheromones from *F. oxysporum*, respectively. VdPpg1 is a Ppg1-like peptide pheromone from *V. dahliae*, VdPpg2 is Ppg2-like peptide pheromone from *V. dahliae*. The dotted circle on the left is a schematic diagram. The pheromone was added to the circular filter paper in the middle, and the strain to be tested was inoculated at the four surrounding sites as illustrated

Similar to the pheromone peptides present in most filamentous fungi, *VdPpg1* encodes a putative pheromone precursor of 212 amino acids containing 6 short tandem repeats of a dodecapeptide sequence, which are always accompanied by the basic dipeptide KR (Kex2-cleavage site) (Fig. 3A). *VdPpg2* encodes a short polypeptide of 66 amino acids with five repeats of a undecapeptide sequence and a CaaX motif at the C-terminus (Fig. 3B). Signal peptide activity assays demonstrated that the signal peptide of VdPpg1 had secretory activity (Fig. 3C). Unlike VdPpg1, VdPpg2 has no predicted signal peptide. Moreover, the pheromone receptor genes *VdPre1* and *VdPre2* of *V. dahliae* were predicted to encode proteins with seven transmembrane domains (Additional file 1: Fig. S2).

To elucidate whether *V. dahliae* can perceive or respond to pheromone, we measured the chemotropic response of the wild-type strains (DK015 and DK038), *MAT* gene deletion strains (Δ*VdMAT1-1-1* and Δ*VdMAT1-2-1*), and the pheromone receptor gene deletion strains (Δ*VdPre1* and Δ*VdPre2*) to the synthesized single-repeat pheromone peptides VdPpg1 and VdPpg2. Considering that *F. oxysporum* and *V. dahliae* have a relatively close evolutionary relationship, the *F. oxysporum* pheromones a-factor and α-factor [60] were also synthesized as controls for the test. With the exception of the responses to the pheromone VdPpg1, all strains exhibited chemotaxis for each of the other types of pheromones. Deletion of mating type genes Δ*VdMAT1-1-1* and Δ*VdMAT1-2-1* had no significant influence on the chemotactic growth of mycelia, while pheromone receptor gene deletion strains (Δ*VdPre1* and Δ*VdPre2*) showed weaker chemotaxis than wild-type strains (Fig. 3D). Since chemotactic growth was anticipated in a direction only towards the pheromone of the opposite mating type strains, further experiments are necessary to clarify this result.

### *VdMAT1-1-1* and *VdMAT1-2-1* impact *V. dahliae* carbon source utilization, stress tolerance, and conidia production

Previous reports indicate that *MAT* genes in filamentous fungi are not only involved in sexual reproduction, but also involved in asexual development, pellet morphology, polar hyphal growth, conidiospore germination, and secondary metabolism [19]. To detect the influence of *VdMAT1-1-1* and *VdMAT1-2-1* on *V. dahliae* vegetative development, the phenotypes of wild-type (DK015 and DK038), knockout (*ΔVdMAT1-1-1* and *ΔVdMAT1-2-1*), and complemented strains (EC^Δ*VdMAT1-1-1*^ and EC^Δ*VdMAT1-2-1*^) of two *MAT* genes were evaluated on media containing different carbon sources and abiotic stressors.

**Fig. 4:**
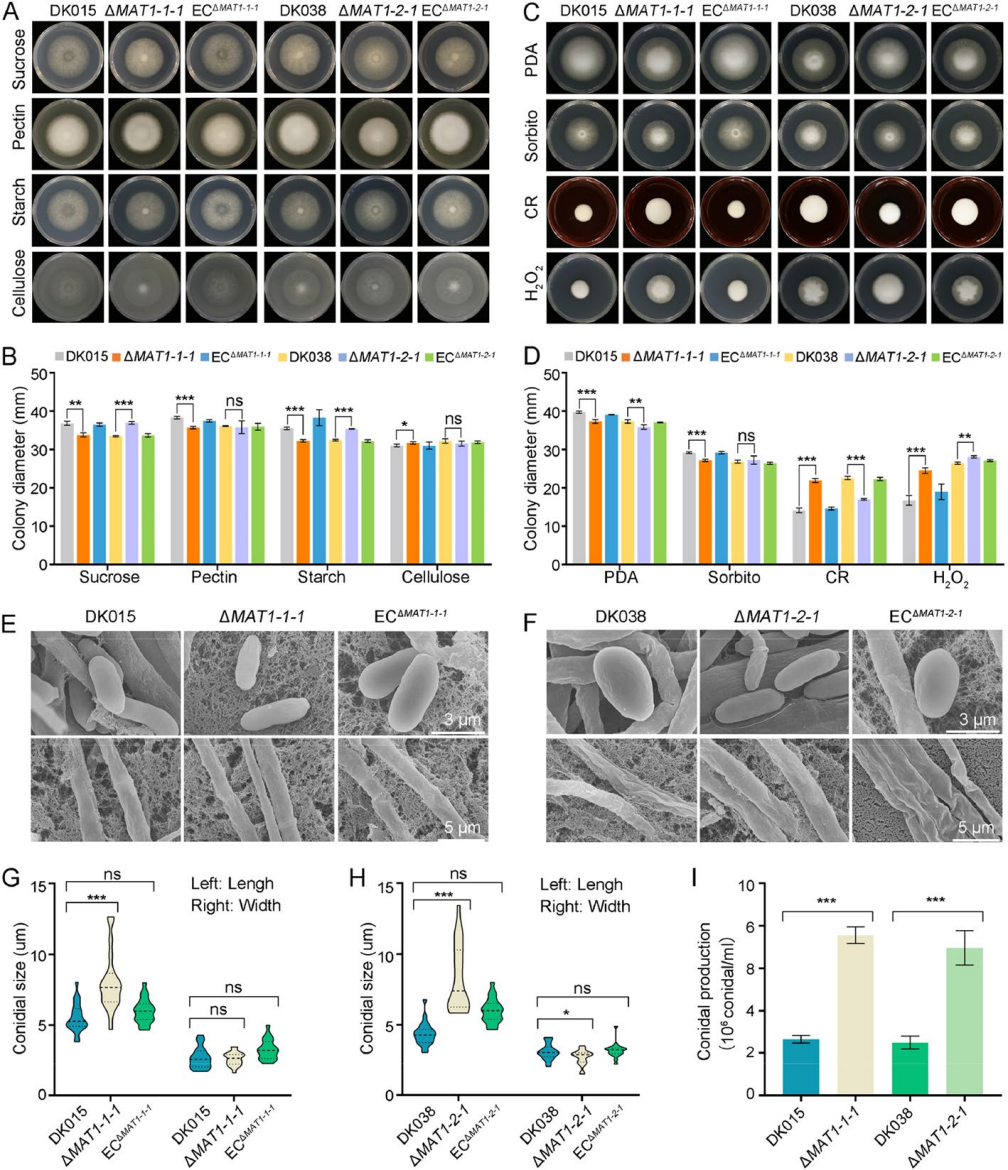
Functional analyses of *VdMAT1-1-1* and *VdMAT1-2-1* in regulating carbon source utilization, stress tolerance, and conidia production of *Verticillium dahliae*. Wild-type strains (DK015 and DK038), *MAT* gene deletion strains (Δ*VdMAT1-1-1* and Δ*VdMAT1-2-1*), and the corresponding complementary strains (EC^Δ*VdMAT1-1-1*^ and EC^Δ*VdMAT1-2-1*^) were used in the experiments. **A**, **B** Growth and diameter (mm) of the colonies of different strains on Czapek medium containing 30 g/L sucrose, 10 g/L pectin, 17 g/L starch, and 10 g/L cellulose, respectively at 25 ℃ for 9 days. **C**, **D** Growth and diameter (mm) of the colonies of different strains on PDA medium containing 1 M sorbitol, 200 μg/ml Congo red, and 2 mM H_2_O_2_, respectively at 25 ℃ for 9 days. **E**, **F** SEM observation of the conidia and mycelia morphology of different strains. **G**, **H** Variation in conidial size. Photographs were taken after 9 days of incubation. **I** Conidial production of different strains. Error bars represent the standard deviation calculated from three replicate experiments compared with the wild-type, and the asterisks show significant differences (one-way ANOVA, *P < 0.05; **P < 0.01; ***P < 0.001)

Compared to the wild-type strain DK015, the colony diameter of the Δ*VdMAT1-1-1* strain was significantly smaller on three media containing sucrose, pectin, and starch as carbon sources (Fig. 4A, B). However, the Δ*VdMAT1-1-1* strain exhibited a larger colony diameter than DK015 on the medium containing cellulose as a carbon source (Fig. 4A, B). The growth phenotype and colony diameter of the complemented strain EC^Δ*VdMAT1-1-1*^ reverted to the phenotypes similar to those of the wild-type strain on the media examined (Fig. 4A, B). On the other hand, the Δ*VdMAT1-2-1* strains grew faster than the wild-type strain DK038 on the media containing sucrose and starch as carbon sources, while there were no evident differences on the media containing pectin or cellulose as carbon sources (Fig. 4A, B). Similarly, there was also no significant difference in growth between EC^*ΔVdMAT1-2-1*^ and DK038 on these media (Fig. 4A, B). Whether *MAT* genes regulate tolerance to abiotic stress was examined subsequently. The ∆*VdMAT1-1-1* strains was more sensitive to sorbitol, but less sensitive to Congo red and H_2_O_2_ compared with wild-type and complemented strains (Fig. 4C, D). The ∆*VdMAT1-2-1* strains were more sensitive to Congo red, but less sensitive to sorbitol and H_2_O_2_ (Fig. 4C, D). The above results indicate divergent responses of the two *MAT* genes for carbon utilization and stress tolerance in the two mating type strains.

To analyze whether *VdMAT1-1-1* and *VdMAT1-2-1* affect the growth of conidia and mycelia in *V. dahliae*, scanning electron microscopy (SEM) was used to observe the morphology of conidia and mycelia. Though deletion of *VdMAT1-1-1* and *VdMAT1-2-1* did not obviously affect the morphology (Fig. 4E, F), the conidia of the Δ*VdMAT1-1-1* and Δ*VdMAT1-2-1* strains were longer than those in the corresponding wild-type strains (Fig. 4G, H). The widths of Δ*VdMAT1-2-1* conidia were also increased compared to that of DK038, while no significant difference was observed between Δ*VdMAT1-1-1* and DK015 in conidial width (Fig. 4G, H). In addition, the conidial yield of Δ*VdMAT1-1-1* and Δ*VdMAT1-2-1* was significantly increased compared to that of the wild-type strains (Fig. 4I). These results indicate a negative regulatory role of the two *MAT* genes in conidia production in two different *MAT* strains.

### *VdMAT1-1-1* and *VdMAT1-2-1* negatively regulate the virulence of *V. dahliae*

**Fig. 5:**
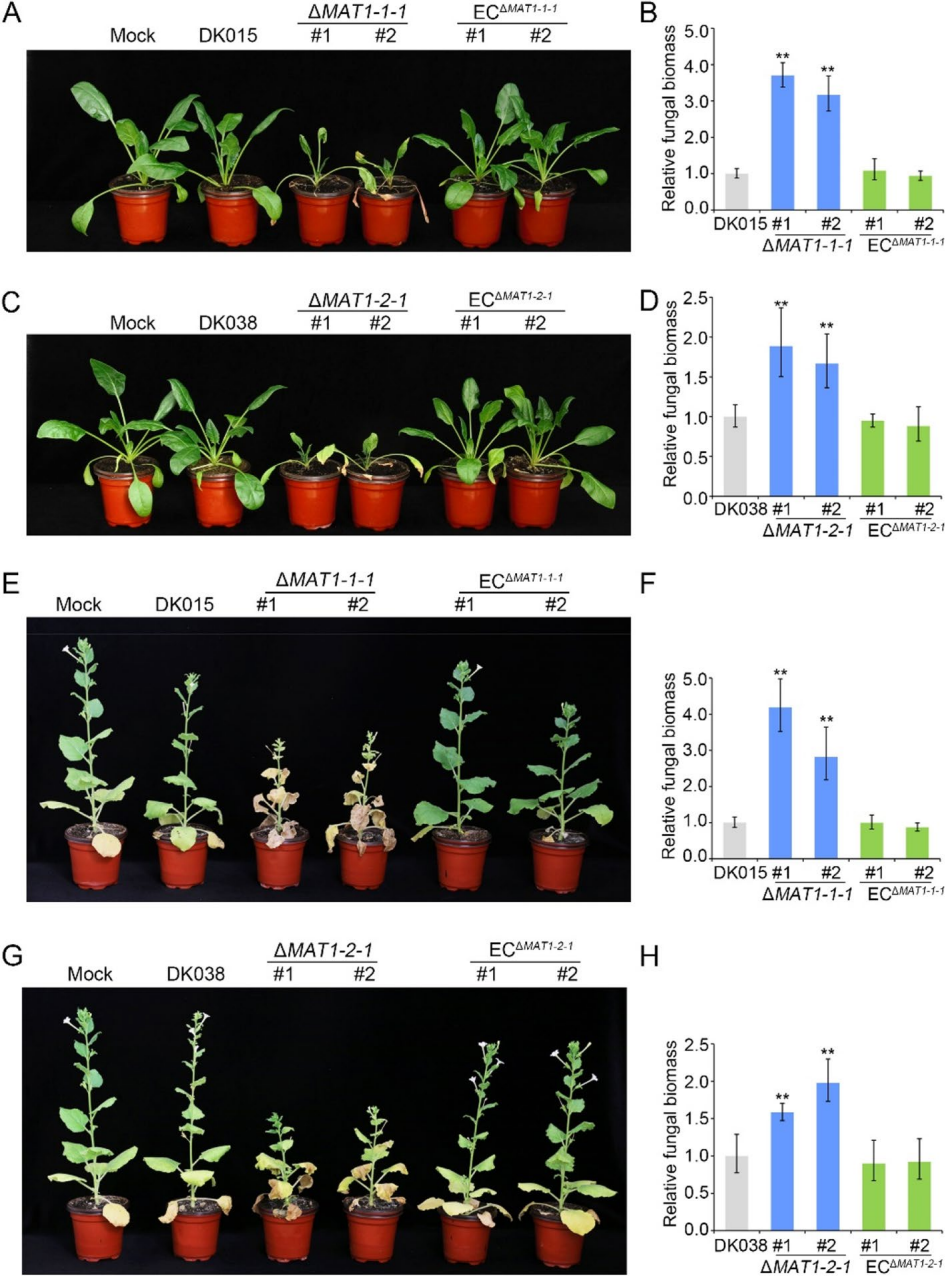
Virulence assays with *VdMAT1-1-1* and *VdMAT1-2-1* deletion mutants and complemented transformant strains of *Verticillium dahliae* on spinach and tobacco. **A**, **B**, **E**, **F** Phenotypes and the fungal biomass of spinach and *N. benthamiana* seedlings inoculated with wild-type strain DK015, Δ*VdMAT1-1-1* mutants, and the corresponding complemented transformants EC^Δ*VdMAT1-1-1*^. **C**, **D**, **G**, **H** Phenotypes and the fungal biomass of spinach and *N. benthamiana* seedlings inoculated with wild-type strain DK038, Δ*VdMAT1-2-1* mutants, and the corresponding complemented transformants EC^Δ*VdMAT1-2-1*^. The fungal biomass was determined by qPCR. *V. dahliae* elongation factor 1-α (*VdEF-1*α) was used to quantify fungal colonization, while the spinach actin gene and the *N. benthamiana EF-1*α gene were used as endogenous plant reference genes, respectively. Error bars represent the standard deviation calculated from three replicate experiments compared with the wild-type. The asterisks show significant differences (one-way ANOVA, **P < 0.01)

To evaluate the roles of *VdMAT1-1-1* and *VdMAT1-2-1* in the pathogenicity of *V. dahliae*, the virulence of the wild-type strain (DK015 and DK038), *MAT* gene deletion strains (Δ*VdMAT1-1-1* and Δ*VdMAT1-2-1*), and the corresponding complemented strains (EC^Δ*VdMAT1-1-1*^ and EC^Δ*VdMAT1-2-1*^) were inoculated on spinach, the host from which they were originally obtained and another host *Nicotiana benthamiana*. The results revealed that Δ*VdMAT1-1-1* and Δ*VdMAT1-2-1* strains increased Verticillium wilt symptoms on these two hosts compared with the wild-type strains DK015 and DK038, which only caused mild disease on spinach and *N. benthamiana* (Fig. 5A, C, E, and G). Correspondingly, the fungal biomass of inoculated spinach and *N. benthamiana* plants with the Δ*VdMAT1-1-1* and Δ*VdMAT1-2-1* strains were significantly increased compared to the wild-type strain (Fig. 5B, D, F, and H). Furthermore, the virulence phenotype or fungal biomass was restored to the level of wild-type strains in the complemented EC^Δ*VdMAT1-1-1*^ and EC^Δ*VdMAT1-2-1*^ strains (Fig. 5A–H). The same results were also obtained using *V. dahliae* strains of different *MAT* loci, such as the *MAT1-1* strain S109 and the *MAT1-2* strain S12, isolated from sunflower (Additional file 1: Fig. S3). These results indicate that *VdMAT1-1-1* and *VdMAT1-2-1* negatively regulate the virulence of *V. dahliae*.

## Discussion

Mating-type genes play important roles not only in regulating the sexual cycle but also in the vegetative growth and pathogenicity of fungi [32, 64, 67]. *V. dahliae*, a heterothallic fungus, has two mating type idiomorphs, *MAT1-1* and *MAT1-2*, located in different strains. In this study, we demonstrated that the *MAT* gene locus of *V. dahliae* was highly conserved, that the genes flanking *MAT1-1* and *MAT1-2* loci have high collinearity (Fig. 1A), and that the core transcription factors VdMAT1-1-1 and VdMAT1-2-1 were localized to the nucleus (Fig. 1C). These MAT proteins play a pivotal role in regulating the expression of pheromone and pheromone receptor genes by directly binding to their respective promoter regions (Fig. 2C, D). *V. dahliae* expresses pheromone precursor genes, with the strains being responsive to pheromones, subsequently exhibiting chemotactic growth (Fig. 3D). In addition, *VdMAT1-1-1* and *VdMAT1-2-1* also play important roles in regulating vegetative growth, stress resistance, and virulence (Figs. 4 and 5). Investigations into the functions of *MAT* genes in *V. dahliae* may facilitate studies to further explore a potential sexual cycle of this destructive pathogen.

One of the most important functions of mating type genes is to regulate the fertility of fungi. Mating-pheromone signaling is required for mate recognition in sexual reproduction of heterothallic fungi [3]. Mating type genes directly control the expression of pheromone precursor and receptor genes, but the regulation mode by which this occurs can differ among fungal species. In *S. macrospora*, the transcription levels of pheromone precursor genes *ppg1* and *ppg2* were significantly downregulated in the Δ*SmtA-1* mutant compared to the wild-type. The expression of *ppg2* was significantly increased in the Δ*SmtA-2* mutant, while the expression of *ppg1* remained unchanged. Additionally, neither mating type proteins SMTA-1 nor SMTA-2 had a clear effect on the expression of pheromone receptor genes *pre1* and *pre2* [33]. In *FusariumF graminearum*, both *MAT1-1-1* and *MAT1-2-1* promoted the expression of pheromone precursor gene *GzPPG1*, while the expression of *GzPPG2* was inhibited by MAT1-1-1 but promoted by MAT1-2-1 [37]. Our results showed that VdMAT1-1-1 and VdMAT1-2-1 promote the expression of pheromone precursor genes *VdPpg1* and *VdPpg2* but inhibit the expression of pheromone receptor genes *VdPre1* and *VdPre2* (Fig. 2A, B). These results indicate that the mechanism by which mating type genes regulate pheromone precursor and receptor genes may be different among fungi and may vary conditionally. Furthermore, pheromones can also induce the expression of mating type genes [12, 16]. This suggests that there is a reciprocal regulation between pheromones and mating type genes to achieve orderly transmission and reception of pheromone signals.

Mate recognition, a critical initial step in sexual reproduction, is mediated in many fungi by the perception of pheromones secreted by partners of the opposite mating type [3]. Our research has demonstrated that the pheromone precursors and receptors in *V. dahliae* exhibit a high degree of similarity in protein sequence to those found in other fungal species (Fig. 3A, B) [7, 37, 42]. Additionally, we have determined that the signaling peptide of VdPpg1 possesses secretory activity (Fig. 3C). Notably, *V. dahliae* displays chemotactic growth in response to mating pheromones, including VdPpg2, FO-a, and FO-α, but not VdPpg1 (Fig. 3D). Initial assays show that *V. dahliae* can undergo chemotactic growth in response to the pheromones of *F. oxysporum*, likely a result of the significant homology between these species [11]. This chemotactic response, however, does not necessarily lead to cell fusion, which could be hindered by heterokaryon incompatibility, akin to a species barrier [50]. The lack of chemotactic growth in response to the synthetic VdPpg1 pheromone raises questions about the congruence between the predicted and actual secreted molecules, a finding that will prompt further investigation into pheromone secretion in *V. dahliae*. Our observations also suggest that both mating types can exhibit chemotaxis towards VdPpg2, a finding that diverges from traditional expectations, such as in *S. cerevisiae*. MATα and MATa secrete α-factor and a-factor, respectively; the α-factor binds to a specific receptor (Ste2) on a MATa cells, whereas the a-factor binds to a specific receptor (Ste3) on MATα cells, which subsequently promotes the chemotactic growth of the two mating strains until fusion [63]. The capacity of *V. dahliae* to grow chemotactically in response to pheromones indicates a conserved communication pathway in its reproductive process, analogous to that observed in known sexually reproducing fungi such as *S. cerevisiae*, *S. macrospora*, and *N. crassa* [31, 42, 63]. However, there may be differences in the mechanisms that regulate pheromone responses in *V. dahliae*. This has prompted us to explore in future work whether *V. dahliae* can secrete pheromones, the specific type of pheromone secreted by *MAT1-1* and *MAT1-2* mating type strains, the corresponding pheromone receptor, the preference of *MAT1-1* and *MAT1-2* strains for different types of pheromones, the signal pathway used for pheromone transmission, and the gene network involved in information exchange between *MAT1-1* and *MAT1-2* strains mediated by pheromones.

Mating type genes are essential for the ascospore formation, mycelial morphology, conidial formation, and stress responses in many fungi [8, 61, 62, 65]. In *V. dahliae*, *VdMAT1-1-1* and *VdMAT1-2-1* are involved in carbon utilization and stress tolerance, but the extent of this involvement differentiates the two mating type strains, especially in vegetative growth (Fig. 4A–D). The conidia of the Δ*VdMAT1-1-1* and Δ*VdMAT1-2-1* mutants became slender (Fig. 4E–H). Similarly, in *Ulocladium botrytis*, *MAT1-1-1* regulates the vegetative growth and the size of the conidia [62]. In *Penicillium chrysogenum*, *MAT1-1-1* controls hyphal morphology and conidia formation [8]. However, in *Villosiclava virens*, the deletion of *MAT1-1-1* resulted in slower growth and abnormal conidial morphology, yet no significant differences were detected in conidial production [65]. These studies, including our work, further suggest that *MAT1-1-1* and *MAT1-2-1* genes have different functions in regulating vegetative growth, asexual reproduction, and responding to external stress in different fungi.

Deletion of either the *VdMAT1-1-1* or *VdMAT1-2-1* genes resulted in a significantly increased pathogenicity (Fig. 5 and Additional file 1: S3). Interestingly, in *F. graminearum*, the virulence of the *MAT1-1-1* gene deletion mutant was reduced on cornstalks [67] and the pathogenicity of the *MAT-2* gene deletion mutant of *Sclerotinia sclerotiorum* was also significantly decreased [17]. In contrast, in *Magnaporthe oryzae*, deletion of *MAT1-1-1* or *MAT1-2-1* genes did not affect appressorium formation and virulence [61]. The functional differences of mating type genes between closely related fungal species indicate the complexity in the network of MAT1-1-1 or MAT1-2-1 protein interactions. The mechanism by which the *VdMAT1-1-1* and *VdMAT1-2-1* genes regulate the virulence of strains is still unclear at present. In subsequent studies, it may be possible to screen differentially expressed genes regulated by mating type genes under host-induced conditions and directly identify the target genes. Analysis of the function of these genes may facilitate exploitation of the mechanism by which mating type genes regulate virulence.

## Conclusions

Mating type genes hold significant importance in fungi, and gaining insights into their molecular functions can substantially aid in unraveling the life cycle of these organisms. While *V. dahliae* is traditionally regarded as a strictly asexual fungus, the function of the mating type genes within this species has not been sufficiently examined. This study shows that *VdMAT1-1-1* and *VdMAT1-2-1* play important roles in asexual reproduction, and perhaps also in sexual reproduction of *V. dahliae*. The findings from this research will serve as a valuable reference for furthering our understanding of sex-related genes and potential mating in *V. dahliae*. In the ongoing follow-up studies, we are exploring whether the two mating type genes can mediate nuclear fusion and sexual reproduction in *V. dahliae*, and the regulatory mechanisms governing sexual reproduction. This work is further expected to illuminate on the mode of cell-cell communication and recognition processes between strains with the opposite mating type idiomorphs, and may offer a novel perspective on the mode of sexual reproduction in *Verticillium* spp. and potentially other ascomycetes.

## Methods

All primers used in this study are listed in Additional file 2: Table S1.

### Bioinformatics analysis

The wild-type strains DK015 and DK038 used in this study were collected from spinach plants exhibiting wilt symptoms. The mating type loci of DK015 and DK038 were identified by the BLASTN using the *VdMAT1-1-1* and *VdMAT1-2-1* from VdLs.17 as the query sequences [58]. The sequences of *VdMAT1-1-1*, *VdMAT1-2-1*, *VdPpg1*, *VdPpg2*, *VdPre1*, and *VdPre2* were cloned from DK015 and DK038. The genomic sequences of these strains were archived in the Verticilli-Omics database (https://db.cngb.org/Verticilli-Omics/), which served as a vital reference for our cloning process.

### Subcellular localization assays

To determine the subcellular localization of VdMAT1-1-1 and VdMAT1-2-1, the fragment of TrpC-promoter region, the coding sequence of *VdMAT1-1-1* or *VdMAT1-2-1*, GFP sequence, and Nos-terminator were fused and cloned into a pCOM vector [68]. The positive recombinant vectors were then introduced into *Agrobacterium tumefaciens* strain AGL-1 for fungal transformation. The positive transformants were selectively cultured on potato dextrose agar (PDA) medium supplemented with 50 µg/mL geneticin. After 7-day culture, conidia were observed using a Leica TCS SP8 confocal microscope. Fluorescent signals were detected at 532 nm excitation and 588 nm emission wavelengths for RFP, 484 nm excitation and 507 nm emission wavelengths for GFP, and 340 nm excitation and 488 nm emission wavelengths for DAPI, respectively.

### *V*. *dahliae* transformations for gene deletion and complementation

Gene deletion vectors of *VdMAT1-1-1*, *VdMAT1-2-1*, *VdPre1*, and *VdPre2* were generated by homologous recombination. In brief, approximately 1.5-kb sequences of the regions flanking the coding sequence of each gene were amplified by PCR using the appropriate primer sets. The plasmid pGKO2 was linearized by restriction endonuclease *EcoR*I or *Hind*III, and the amplified up- and downstream fragments were ligated with the hygromycin resistance gene cassette (hyg). To generate the vector for mutant complementation, the genomic sequence of each gene, including its native promoter, coding region, and terminator were amplified by PCR using the appropriate primer sets, and then fused into the pCOM vector which carries geneticin resistance cassette (*G418*) [68].

The *A. tumefaciens* mediated transformation (ATMT) method, as previously described, was employed to generate gene deletion and complementation transformants [40]. The positive gene deletion strains were selected on PDA medium supplemented with 50 μg/mL hygromycin, 200 µg/mL cefotaxime, and 200 μg/mL 5-fluoro-2’-deoxyuridine. The complemented strains were selected on PDA medium supplemented with 50 µg/mL geneticin. All positive strains were further verified by conducting PCR to amplify the corresponding specific sequence, using the appropriate primer sets as listed in Additional file 2: Table S1.

### Yeast signal sequence trap system

Functional validation of the signal peptide of VdPpg1 was performed as previously described [28]. The sequence encoding the predicted signal peptide of *VdPpg1* was cloned into the pSUC2 vector. The resulting recombinant plasmid, pSUC2::SP^VdPpg1^, was transformed into the yeast strain YTK12 and screened on CMD-W medium.

The recombinant YTK12 strain carrying the signal peptide sequence of *Avr1b* (pSUC2::SP^Avr1b^), and the untransformed YTK12 strain and YTK12 strain with an empty pSUC2 vector were used as a positive control and negative controls, respectively. These strains were incubated in a 10 mM acetic acid-sodium acetate solution (pH = 4.7) and 10% sucrose medium for 10 min at 37 °C. Post-incubation, the supernatant was collected and incubated with 0.1% 2,3,5-triphenyltetrazole ammonium chloride (TTC) for 10 min. Invertase enzymatic activity was confirmed by observing an increase in insoluble red-colored triphenyl formazan, indicating the reduction of TTC and signifying successful signal peptide functionality.

### Verification of hyphal chemotropism

To verify hyphal chemotropism, pheromones of VdPpg1 (GKPCPRPGQGCW), VdPpg2 (NGGGHSGCAIM), *Fo*-α (WCTWRGQPCW), and Fo-a (ANGQTPGYPLSCTVM) were chemically synthesized and dissolved in 50% (v/v) methanol (MeOH) to a concentration of 378 μM [60]. For the assay, 25 μL of the pheromone solution was applied to a 1.2-cm-diameter piece of filter paper, which was then placed at the center of water agar medium plates. A 3 μL conidial suspension, with a concentration of 5 × 10^6^ conidia/mL of the different strains, was cultured on water agar medium plates and positioned 1.2 cm away from the filter paper. The plates were incubated for 7 days at 25 °C to observe chemotropic responses.

### Dual-luciferase report assay

The coding regions of *VdMAT1-1-1* and *VdMAT1-2-1* were cloned and fused into the pCAMBIA1300-Cluc vector [9] to generate the effector constructs. Meanwhile, the promoter regions of *VdPpg1*, *VdPpg2*, *VdPre1*, and *VdPre2* were introduced into the pGreenII 0800-LUC vector [26], each generating a distinct reporter construct. Both recombinant vectors were transformed into *A. tumefaciens* strain GV3101 cells. The firefly luciferase and *Renilla* luciferase activities were analyzed at 60 hpi using the Dual-Luciferase Reporter Assay System (Promega) and the GloMax96 Microplate Luminometer (Promega). Each experiment was performed with three biological replicates.

### Electrophoretic mobility shift assay (EMSA)

The full coding regions of *VdMAT1-1-1* and *VdMAT1-2-1* were amplified from the cDNA of the DK015 and DK038 strains, respectively. These amplified sequences were inserted into the *EcoR*I/*Sal*I-digested pGEX-4T-1 vector by homologous recombination of multiple fragments (ClonExpress Ultra One Step Cloning Kit, Vazyme, Nanjing, China), to generate the prokaryotic expression vectors pGEX-*VdMAT1-1-1* and pGEX-*VdMAT1-2-1*. Positive recombinant vectors were transformed into the *Escherichia coli* BL21(DE3) strain. VdMAT1-1-1 and VdMAT1-2-1 proteins were purified following the instructions of GST-tag protein purification kit (Beyotime, Shanghai, China). For DNA shift assays, the promoter fragments of the target genes were labeled using 6-carboxyfluorescein (FAM). The assays were performed with the EMSA binding buffer kit, following the instructions of the manufacturer (Beyotime, Shanghai, China).

### Yeast one-hybrid assays

Yeast one-hybrid assays were performed using the Matchmaker Gold Yeast One-Hybrid System Kit (Takara) according to the manufacturer’s protocol. Briefly, gene (*VdPpg1*, *VdPpg2*, *VdPre1*, and *VdPre2*) promoter fragments were ligated into the pAbAi vector, while the full-length CDS of *VdMAT1-1-1* and *VdMAT1-2-1* were cloned into the pGADT7 vector (gene-AD). The gene-AD vectors were then used to transform Y1HGold cells harboring the pAbAi-bait and then screened on SD/-Leu/AbA medium.

### Strains growth, stress response, and conidiation assays

The strains were routinely cultured on PDA medium or maintained in a shaking incubator using complete medium (CM) for 5 days at 25 °C in the dark. For phenotype analysis, 3 μL of the conidial suspension with a concentration of 5 × 10^6^ conidia/mL was cultured on Czapek plates (FeSO_4_, 0.01 g/L; KCl, 0.5 g/L; MgSO_4_·7H_2_O, 0.5 g/L; K_2_HPO_4_, 1 g/L; NaNO_3_, 3 g/L; and agar, 18 g/L) prepared with different carbon sources: either sucrose, 30 g/L; pectin 10 g/L, starch, 17 g/L, or cellulose, 10 g/L.

For stress response assays, 3 μL of the conidial suspensions, each with a concentration of 5 × 10^6^ conidia/mL, was cultured on PDA medium, supplemented with either 1 M sorbitol, 200 μg/mL Congo red, or 1.5 mM H_2_O_2_, each prepared separately. After 7 days of incubation at 25 °C, the phenotypes including colony diameters were observed.

To measure the sporulation of *V. dahliae*, 3 μL of each conidial suspension with a concentration of 5 × 10^6^ conidia/mL was cultured on PDA plates for 9 days at 25 °C. Three same areas of the fungus were collected and suspended in 1mL of sterile water by vortex for 1 min. The numbers of conidia were counted using a hemocytometer.

### Virulence assays

Six-week-old spinach and 5-week-old tobacco seedlings were inoculated by the root irrigation method [23]. Each pot was inoculated with 50 mL of the conidial suspension with a concentration of 5 × 10^6^ conidia/mL. Plants were maintained on the greenhouse benches at 24 ± 2 °C under a 16-h photoperiod after inoculation. The phenotypes of spinach seedlings were investigated 30 days after inoculation, while those of tobacco seedlings were evaluated on the 21^st^ day. For molecular analysis, the root-stem junctions of spinach and tobacco plants were collected and DNA was extracted. Biomass of *V. dahliae* was quantified by qPCR following the procedure of Santhanam et al. [49]. The *V. dahliae* elongation factor 1α (*VdEF-1α*) was used to quantify fungal colonization and the spinach *actin* gene and *N. benthamiana EF-1α* gene were used as endogenous reference genes.

### Supplementary Information

**Additional file 1: Figure S1. ***MAT1-1* and *MAT1-2* loci and flanking sequences in the *MAT1-1* and the *MAT1-2 *strains isolated from different hosts. **Figure S2.** Schematic diagram of the transmembrane domains in VdPre1 and VdPre2 proteins predicted by TMHMM. **Figure S3.** Virulence assays with *VdMAT1-1-1* and *VdMAT1-2-1 d*eletion mutant of *Verticillium dahliae* on sunflower.

**Additional file 2: Table S1.** Information on the primer pairs used to construct the vector in this study.

**Additional file 3.** Raw data of experimental results.

## Data Availability

All study data are included in the article and/or supplementary information.
