# Supplementary material for: Functional analysis of the mating type genes in Verticillium dahliae
Source: BMC Biol. 2024 May 7;22:108. doi: 10.1186/s12915-024-01900-6 (PMC11077750; doi:10.1186/s12915-024-01900-6)
Supplement: Supplementary file 1 — Additional file 1: Figure S1. MAT1-1 and MAT1-2 loci and flanking sequences in the MAT1-1 and the MAT1-2 strains isolated from different hosts. Figure S2. Schematic diagram of the transmembrane domains in VdPre1 and VdPre2 proteins predicted by TMHMM. Figure S3. Virulence assays with VdMAT1-1-1 and VdMAT1-2-1 deletion mutant of Verticillium dahliae on sunflower. [file 12915_2024_1900_MOESM1_ESM.doc]

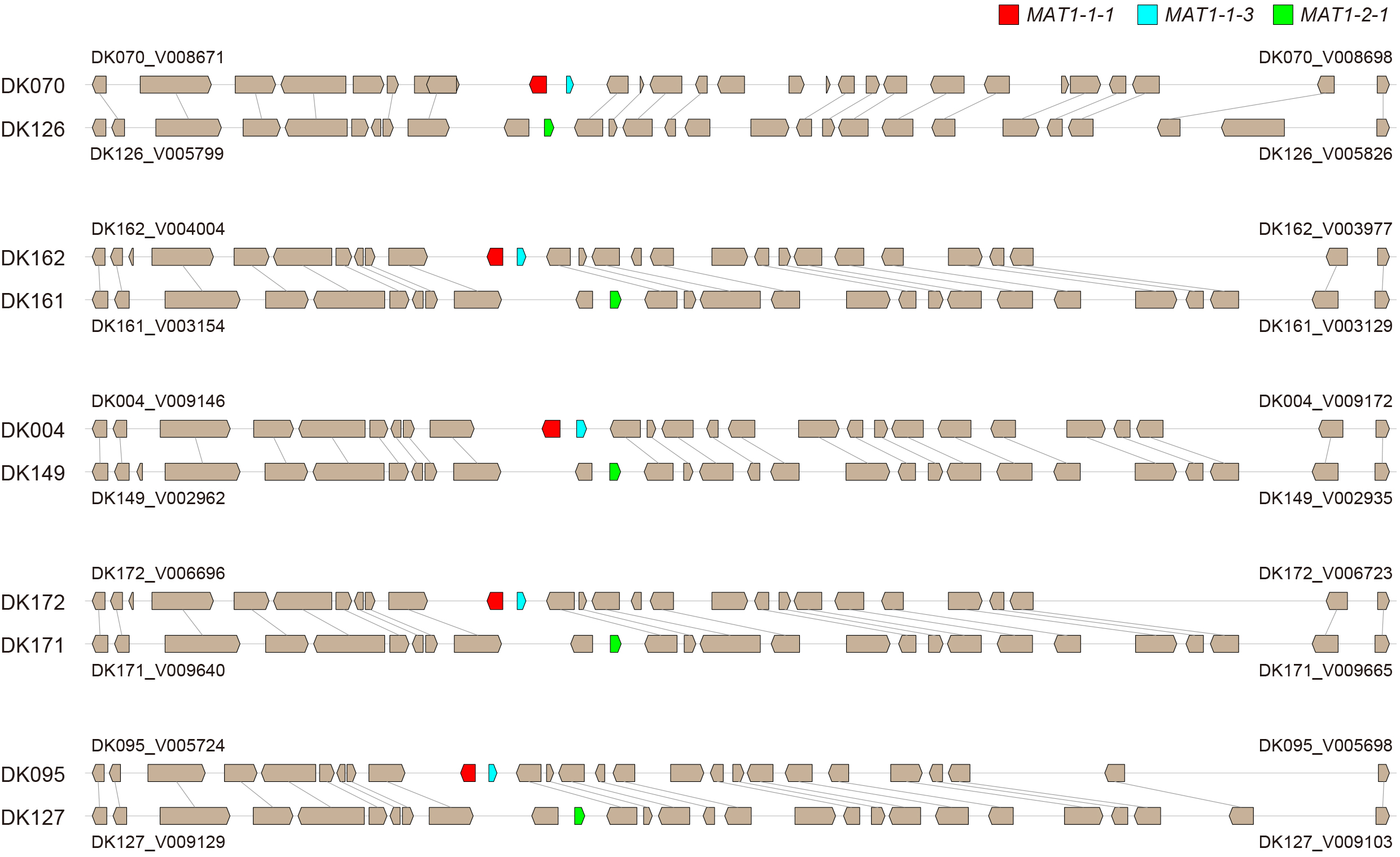


**Fig. S1 Diagram of *MAT1-1* and *MAT1-2* loci and flanking sequences in the *MAT1-1* and the *MAT1-2* strains isolated from different hosts.**

Rectangles represent the genes within the two loci. The dashed line indicates the syntenic relationship between two homologous genes flanking both loci. Gene IDs are marked for the first and last gene marked. Red and bright light blue rectangles indicate genes specific to *VdMAT1-1* locus, *VdMAT1-1-1* and *VdMAT1-1-3*, respectively, while the green rectangle indicates the gene specific to *VdMAT1-2* locus, *VdMAT1-2-1*. The five combined *MAT1-1* and *MAT1-2* strains, DK070 and DK126, DK162 and DK161, DK004 and DK149, DK172 and DK171, as well as DK095 and DK127, were isolated from tomato, potato, cotton, sunflower, and watermelon respectively.


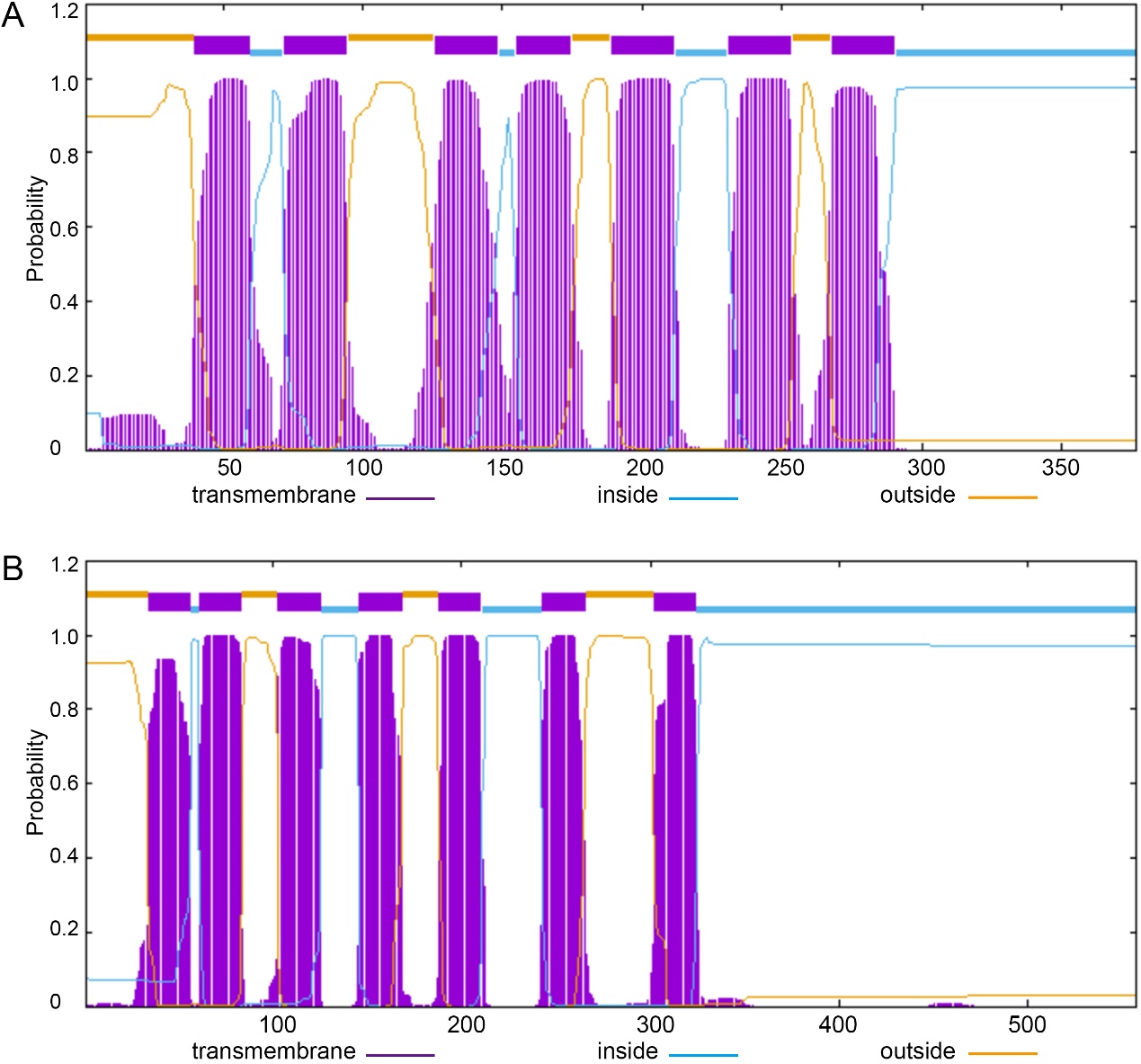


**Fig. S2 Schematic diagram of the transmembrane domains in VdPre1 and VdPre2 proteins predicted by TMHMM. A and B.** Display of the transmembrane domains in VdPre1 and VdPre2, respectively.


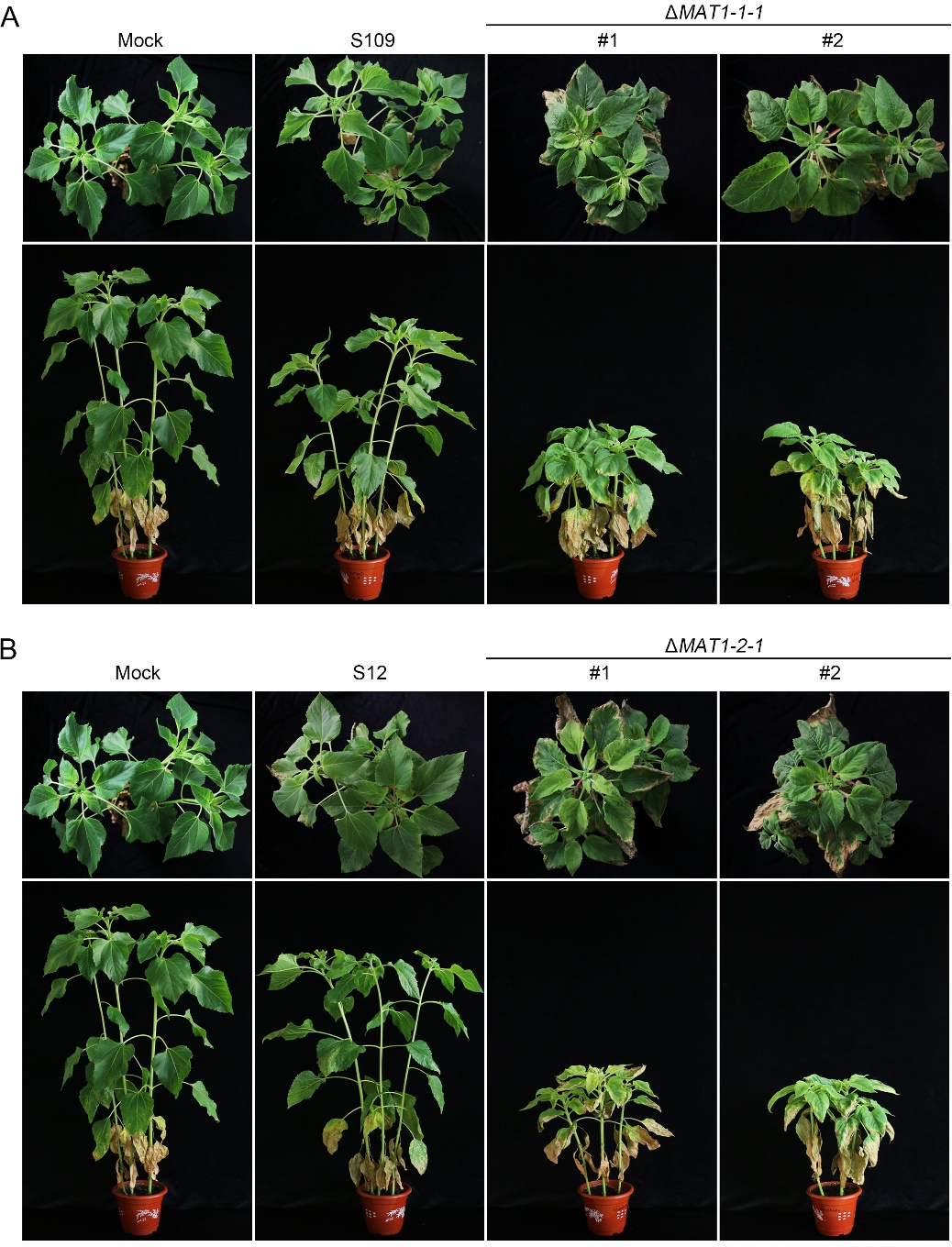


**Fig.** **S3 Virulence assays with *VdMAT1-1-1* and *VdMAT1-2-1* deletion mutants of *Verticillium dahliae* on sunflower. A.** Phenotypes of sunflower seedlings inoculated with wild type strain S109 and its corresponding Δ*VdMAT1-1-1* mutants. **B.** Phenotypes of sunflower seedlings inoculated with wild type strain S12 and its corresponding Δ*VdMAT1-2-1* mutants.
